# Genotype-by-diagnosis interaction influences self-control in human cocaine addiction

**DOI:** 10.1038/s41398-023-02347-z

**Published:** 2023-02-11

**Authors:** Michal M. Graczyk, Barbara J. Sahakian, Trevor W. Robbins, Karen D. Ersche

**Affiliations:** 1 Department of Psychiatry, University of Cambridge, Cambridge, UK; 2 Department of Psychology, University of Cambridge, Cambridge, UK; 3 Department of Systems Neuroscience, University Medical Center Hamburg-Eppendorf, Hamburg, Germany; 4 Department of Addictive Behaviour and Addiction Medicine, Central Institute of Mental Health, University of Heidelberg, Mannheim, Germany

**Keywords:** Addiction, Human behaviour

## Abstract

Not everyone who uses drugs loses control over their intake, which is a hallmark of addiction. Although familial risk studies suggest significant addiction heritability, the genetic basis of vulnerability to drug addiction remains largely unknown. In the present study, we investigate the relationship between self-control, cocaine use, and the *rs36024* single nucleotide polymorphism of the noradrenaline transporter gene (*SLC6A2*). We hypothesize that C-allele-carrying adults show impaired self-control, as measured by the stop-signal task and demonstrated previously in adolescents, and further exacerbated by chronic cocaine use. Patients with cocaine use disorder (CUD, *n* = 79) and healthy unrelated participants with no history of drug abuse (*n* = 54) completed the stop-signal task. All participants were genotyped for *rs36024* allelic variants (CC/TT homozygotes, CT heterozygotes). We measured mean stop-signal reaction time, reflecting the ability to inhibit ongoing motor responses, reaction times to go stimuli, and the proportion of successful stops. CUD patients showed prolonged stop-signal reaction time, however, there was no main effect of *rs36024* genotype. Importantly, there was a significant genotype-by-diagnosis interaction such that CUD patients with CC genotype had longer stop-signal reaction time and fewer successful stops compared with CC healthy controls and TT CUD patients. CT CUD patients showed an intermediate performance. Self-control deficits were associated with cocaine use disorder diagnosis, which interacts with the noradrenaline transporter *rs36024* polymorphism. Our findings suggest that *rs36024* may represent a potential genetic vulnerability marker, which facilitates the transition from first cocaine use to addiction by weakening the inhibitory control over behavior.

## Introduction

Cocaine use is a growing public health concern, with estimated 20 million users worldwide [1]. Cocaine addiction is a chronically relapsing disorder, characterized by a loss of control over drug use, which develops in staged clinical transitions from first drug use to addiction onset. However, not everyone who uses cocaine develops cocaine addiction, suggesting that the drug interacts with a person’s vulnerability profile. A family history of addiction has been shown to increase the likelihood of developing addiction by an eight-fold [2], which points towards a genetic predisposition. Genome-wide and candidate gene studies have identified several polymorphisms that have been associated with cocaine addiction [3], but it remains unclear how these are implicated in addictive behavior. Endophenotypes have been proposed as a strategy to enhance the power of quantitative trait locus approaches to identify risk genes that predispose complex genetic disorders [4, 5]. Previous research using an endophenotype approach revealed that self-control abilities, as measured by the stop-signal task [6], were not only impaired in patients with stimulant use disorder but also in their unaffected biological siblings [7]. Prolonged stopping performance in these sibling pairs was further associated with reduced white matter integrity in the right inferior frontal gyrus, a brain region critically implicated in self-control [8]. This suggests that weak inhibitory control may have pre-dated drug-taking and potentially rendered individuals who used stimulant drugs vulnerable for developing addiction. Neuroimaging work further revealed that the unaffected siblings were able to compensate for their vulnerability by over-activating the inhibitory control network during stop-signal task performance [9]. Such a compensatory response was, however, not seen in their addicted siblings, suggesting that the ability to increase stopping capacity might have been impaired by chronic use of stimulant drugs.

Self-control abilities vary considerably within the normal population. Accumulating evidence points towards genetic polymorphisms in monoamine neuromodulator systems underlying brain and behavioral stop-signal performance, including stop-signal reaction time [10–13], inter-individual variation in response times [14] and task-related brain activation [10, 13, 15]. A large multicenter study investigated 1,593 adolescents’ stop-signal performance twice, at the age of 14 and 16 years [15]. They found that the C allelic variant of *rs36024* single nucleotide polymorphism in noradrenaline transporter gene (*SLC6A2*) was associated with reduced task-related activation in brain networks implicated in stopping performance on the stop-signal task in those adolescents who were most likely to use the drugs at the age of 16 years. The authors identified *rs36024* as a potential candidate genetic risk marker for addiction vulnerability, implicated in response-inhibition. This proposal receives support from reports of improved stop-signal performance following the inhibition of central noradrenaline reuptake in both patients and healthy volunteer samples [16, 17]. However, some studies did not replicate these findings [18, 19], suggesting individual variation in response to noradrenaline reuptake inhibitor atomoxetine and thus differential extracellular noradrenaline concentrations during the stop-signal task.

Here, we sought to investigate the influence of the *rs36024* polymorphism on stop-signal performance in people with and without cocaine use disorder (CUD). Adults with cocaine addiction have been repeatedly shown to have prolonged stop-signal reaction time [20–25] and may thus benefit most from pharmacological interventions to improve self-control [26–28]. We hypothesize that self-control, as reflected by stop-signal reaction time, is specifically impaired in the carriers of *rs36024* C-allele, irrespective of whether they use cocaine or not. We further hypothesize that a CUD diagnosis interacts with a genetic vulnerability, impairing stop-signal performance even further. The latter would to the best of our knowledge constitute the first demonstration of genotype-by-diagnosis interaction in impairing behaviors that define the addiction phenotype.

## Methods

### Study sample

We recruited a total of 133 participants (92% male) from the local community in Cambridge (UK) by advertisement and by word-of-mouth. Participants had to be at least 20 years of age, in good general health, and proficient in English. All participants underwent a medical review and a psychiatric screening using the Mini International Neuropsychiatric Inventory [29]. Drug use was further assessed using the Structured Clinical Interview for DSM-IV-TR [30]. Seventy-nine participants (91% male) met the diagnostic criteria for cocaine dependence according to the DSM-IV-TR [31], thence referred to as Cocaine Use Disorder (CUD). On average, they started using cocaine at the age of 20.6 years [standard deviation (SD) ± 5.5 years] and have been actively using the drug for 15.8 years (SD ± 6.7). Sixty-seven percent of CUD patients also met the criteria for opioid dependence, but were controlled on either methadone (62%) or buprenorphine (26%). Thirty-five percent of CUD patients met the DSM-IV-TR criteria for cannabis dependence and 6% for alcohol dependence. The remaining 54 participants (94% male) had no personal history of substance dependence, which was also reflected by low scores on the Drug Abuse Screening Test [32] and the Alcohol Use Disorders Identification Test [33] respectively. Six percent of control participants were smoking tobacco and 49% reported a history of sporadic use of cannabis. Exclusion criteria, which applied to all participants included a history of psychotic or neurodevelopmental disorder, a traumatic brain injury, and for healthy volunteers also the use of psychoactive medication. Unrelated data of the sample have been published previously [34–36].

### Study procedures

Prior to testing, all participants were breathalyzed to verify that they were not intoxicated by alcohol and urine samples were screened for undeclared drugs; all samples provided by CUD patients tested positive for cocaine and all samples provided by control participants tested negative. Vital signs (blood pressure and pulse rate) were taken before a blood sample was drawn for genotyping. LGC Genomics Ltd. (www.lgcgroup.com/genomics) was commissioned to identify the *SLC6A2* gene *rs36024* single nucleotide polymorphism (SNP). All except six participants had their genes extracted and were grouped according to their *rs36024* allelic variant (homozygous CC, heterozygous CT, homozygous TT). All participants provided written informed consent to participate in the study and completed the National Adult Reading Test [37] to estimate verbal intelligence and the Barratt Impulsiveness Scale (BIS-11 [38], to assess trait impulsivity. The Childhood Trauma Questionnaire was administered to assess levels of childhood adversity [39]. All study procedures were approved by the National Research Ethics Committee (12/EE/0519, PI: KD Ersche).

### Stop-signal task

All participants completed the stop-signal task from the Cambridge Cognition Neuropsychological Test Battery (CANTAB, Cambridge Cognition Ltd., Cambridge, UK). They were instructed to press the left and right key on the button box in response to a left and right pointing white arrow (Go-stimuli), which were appearing in succession on the computer screen. However, when an arrow was followed by an auditory tone (100 ms, 300 Hz), participants were told to inhibit responding. The direction of the arrows was intermixed and counterbalanced. According to Logan and colleagues [6], the time between onset of the Go-stimuli and the stop-signal, i.e. the stop-signal delay, was at first set to 250 ms but was thereafter adjusted in 50 ms increments to achieve 50% successful stopping performance for each participant individually (no maximum or minimum values of stop-signal delay were imposed). The task comprised a training block of 16 Go-trials, followed by a total of 240 Go-trials and 80 Stop-trials across five experimental blocks. Go and Stop-trials were randomized within each block. The inter-trial intervals fell in the range of 900 to 1,100 ms. Throughout the task, participants were reminded to respond as quickly as possible and not to wait for the stop-signal to occur. Visual and verbal performance feedback was provided at the end of each experimental block.

Our outcome measure of interest was the mean stop-signal reaction time (SSRT), i.e. the time that participants need to inhibit a prepotent response. SSRT was calculated using the integration method with the replacement of Go omissions, which according to Verbruggen and colleagues [40] has been shown to produce less biased estimates than other methods such as mean- and integration-based methods. According to the independent race model [41] between the Stop- and Go-responses the probability of responding on stop trials should be close to 0.50 [6]. Previous studies applied various cut-off ranges for the exclusion of participants i.e. a liberal range of 0.25–0.75 [40] or a more conservative range of 0.40–0.60 [9, 42]. Applying the latter would result in the exclusion of 40 participants (32 CUD patients, 8 controls; 31% of the sample). To maximize statistical power in the present three-by-two factorial design, we instead used marginally lower cut-off values of 0.35 and 0.65 which is a threshold more conservative than that suggested by the expert consensus [40]. This meant that 18 participants (12 CUD patients, 6 controls) had to be excluded, leaving a total of 111 genotyped participants (63 CUD patients, 48 controls). We also tested the race model by comparing participants’ mean response time (RT) on successful Go with those on unsuccessful Stop trials. Faster RTs on unsuccessful Stop trials relative to Go trials suggest that Go stimuli won the race [9]. Finally, we measured participants’ mean RT after unsuccessful Stop trials, the percentage of successful stops and Go omissions.

### Statistical analysis

Demographic and clinical data were analyzed using one-way analysis of variance to evaluate differences between group and genotype. Unless stated otherwise, behavioral data were analyzed using an univariate analysis of co-variance (ANCOVA) with two fixed factors (diagnostic group, genotype). Group had two levels (control, CUD) and genotype three levels (CC, CT, TT). Both age and mean GoRT were included as covariates to control for group differences in these two variables, as these are known confounds on stop-signal performance [43]. Sidak correction was applied for *post-hoc* tests. All statistical tests were two-tailed and significance levels were set at 0.05. Genotype distribution was analyzed using chi-squared test (*χ*^*2*^). All data were analyzed using the Statistical Package for Social Sciences version 28.0 (IBM SPSS).

## Results

### Demographics, questionnaire and clinical information

Demographic, questionnaire and clinical data with respect to diagnostic group and genotype are shown in Table 1. Genotypes were evenly distributed across the two diagnostic groups (χ^2^_2_ = 1.33, *p* = 0.514) as well as across CUD patients with and without comorbid opiate use disorder (χ^2^_2_ = 0.581, *p* = 0.0.748) and cannabis use disorder (χ^2^_2_ = 3.13, *p* = 0.209). Age did not differ between genotypes (F_2,108_ = 1.77, *p* = 0.175) but significantly between diagnostic groups (F_1,109_ = 4.10, *p* = 0.045), such that CUD patients were significantly younger than controls. To avoid confounding effects of age on stop-signal performance [44], age was included as a covariate. Although group (F_1,107_ = 63.1, *p* < 0.001) but not genotype (F_2,105_ = 0.678, *p* = 0.510) differed with respect to verbal intelligence, this has not been considered a confounding factor for stop-signal performance [45]. Self-reported impulsivity (as assessed by the BIS-11 total score) was significantly increased in CUD patients (F_1,110_ = 85.3, *p* < 0.001) but not across genotypes (F_2,110_ = 0.872, *p* = 0.421), and was not included as a covariate because impulsivity is a defining characteristic of CUD, and therefore should not be statistically controlled for [46]. Alcohol consumption did not differ between CUD patients and control participants (F_1,110_ = 0.011, *p* = 0.918) or between genotypes (F_2,108_ = 0.055, *p* = 0.946). CUD patients reported higher levels of childhood adversity (F_1,84_ = 16.5, *p* < 0.001), which did not differ across genotypes (F_2,84_ = 2.23, *p* = 0.114). Subgroup comparisons of demographics and questionnaire data between CUD patients with and without opiate use disorder and CUD patients with and without cannabis use disorder did not reveal significant differences (see Tables S1, S2).

**Table 1:** Demographics, personality traits and clinical data [mean and standard deviation, (Std.)] presented by group and genotype.

	Genotype (rs36024)	CC		CT		TT	
	Clinical Status	Mean	Std.	Mean	Std.	Mean	Std.
Sample size (n)	Control participants	23	–	12	–	13	–
	CUD patients	25	–	22	–	16	–
Age (years)	Control participants	41.9	10.3	43.4	9.6	42.4	12.2
	CUD patients	36.0	7.9	38.6	8.0	43.4	8.5
Gender (% male)	Control participants	91%	–	100%	–	92%	–
	CUD patients	88%	–	95%	–	88%	–
Verbal intelligence (NART score)	Control participants	114.9	6.8	115.9	6.5	113.2	7.9
	CUD patients	101.1	8.6	100.2	7.2	105.7	9.3
Impulsivity trait (BIS-11, total score)	Control participants	59.7	7.8	58.3	7.9	60.4	5.3
	CUD patients	76.0	10.0	76.0	10.6	73.4	8.1
Drug use experiences (DAST-20, total score)	Control participants	0.4	0.6	0.2	0.4	0.6	0.8
	CUD patients	–	–	–	–	–	–
Childhood maltreatment (CTQ, abuse score)	Control participants	16.2	1.8	16.6	2.4	19.3	9.2
	CUD patients	23.5	9.0	24.6	13.9	32.0	15.5

### Behavioral results

In keeping with the assumptions of the race model, RTs on unsuccessful Stop trials were faster compared with Go trials in both diagnostic groups (CUD patients t_76_ = 4.66, *p* < 0.001; controls t_52_ = 11.9, *p* < 0.001; Table 2). No participant was excluded due to prolonged reaction times. CUD patients had significantly longer RTs on Go trials (GoRT) compared with controls (F_1,110_ = 17.0, *p* < 0.001), but there was no main effect of genotype (F_2,110_ = 0.501, *p* = 0.607), and no genotype-by-diagnosis interaction (F_2,110_ = 1.84, *p* = 0.163). To statistically control for the overall slowing of CUD patients (which might include individuals who slowed responses strategically), GoRT was included as a covariate in all further analysis [43].

**Table 2:** Participant performance on the stop-signal task according to clinical group and genotype.

	Genotype (rs36024)	CC		CT		TT	
	Clinical Status	Mean	Std.	Mean	Std.	Mean	Std.
RT on Go trials (ms)	Control participants	364.0	52.6	384.4	54.9	365.3	51.2
	CUD patients	471.4	114.2	415.1	72.5	439.4	127.0
SSRT (ms)	Control participants	169.8	42.8	185.9	39.2	181.2	56.1
	CUD patients	212.2	64.5	187.2	59.8	182.5	56.2
Stop success (%)	Control participants	43.6	4.3	43.9	4.2	41.5	4.0
	CUD patients	44.2	5.2	44.5	5.4	46.5	6.4
RT on unsuccessful Stop trials (ms)	Control participants	328.4	40.3	330.1	32.1	323.9	35.3
	CUD patients	368.3	57.6	353.6	46.9	365.8	68.0
RT after unsuccessful Stop trials (ms)	Control participants	391.7	60.4	421.8	63.1	382.3	44.6
	CUD patients	494.9	126.8	428.4	72.1	469.4	120.5
Go omissions (%)	Control participants	2.4	2.5	2.6	1.5	2.8	3.1
	CUD patients	4.7	4.6	4.2	2.8	2.9	3.0

Mean and standard deviation (Std.).

*RT* response time, *SSRT* stop-signal reaction time.

**Fig. 1:**
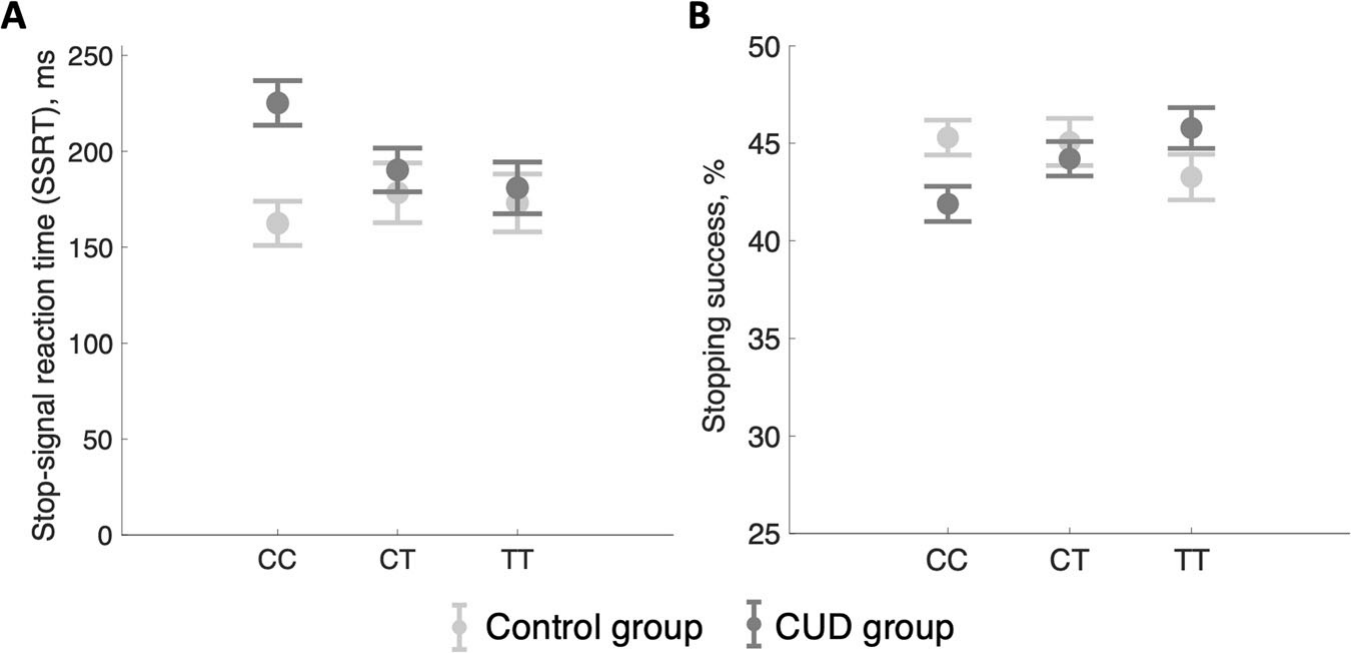
Stop-signal task performance as per diagnostic group (control and CUD) and rs36024 SNP genotype (CC, CT and TT on *x*-axis). **A** SSRT was differently affected by genotype and diagnostic group, such that CUD patients with CC genotype showed significantly prolonged SSRT compared with CC control participants and TT CUD patients. **B** The percentage of successful stops was also differently affected by genotype and diagnostic group, such that CUD patients with CC genotype had fewer successful stops compared with both CC control participants and TT CUD patients. Error bars denote ±standard error of the mean.

SSRT significantly differed between the diagnostic groups (F_1,110_ = 5.62, *p* = 0.020) but not between genotypes (F_2,110_ = 0.897, *p* = 0.411). We also observed a significant genotype-by-diagnosis interaction (F_2,110_ = 3.16, *p* = 0.047), suggesting that the prolonged stopping response in CUD patients was differentially affected by genotype. Indeed, as shown in Fig. 1A, CUD patients with homozygous CC *rs36024* SNP had significantly longer SSRT compared with CC controls (F_1,103_ = 13.3, *p* < 0.001) and TT CUD patients (F_2,103_ = 3.816, *p* = 0.041). Importantly, overall stopping performance (as reflected by the percentage of successful stops) was not significantly different between the diagnostic groups (F_1,110_ = 0.420, *p* = 0.519) or genotypes (F_2,110_ = 0.742, *p* = 0.478), but again, stopping performance was differentially affected by genotype in CUD patients and controls, as reflected by a significant genotype-by-diagnosis interaction (F_2,110_ = 4.47, *p* = 0.014). As shown in Fig. 1B, CUD patients with homozygous CC showed fewer successful stops compared with their CC counterparts in the control group (F_1,103_ = 6.50, *p* = 0.012) or with TT CUD patients (F_2,103_ = 4.22, *p* = 0.017). A subgroup analysis between CUD patients with and without co-morbid opiate use disorder did not reveal any significant differences in performance, nor did the subgroup analysis between CUD patients with and without co-morbid cannabis use disorder (Fig. S1). RT on unsuccessful Stop trials, which reflects participants’ intention to stop, were not significantly different between groups (F_1,110_ = 0.002, *p* = 0.960) or genotypes (F_2,110_ = 0.172, *p* = 0.842; Table 2). There was a marginally significant genotype-by-diagnosis interaction (F_2,110_ = 3.08, *p* = 0.050), which appeared to be driven by CUD patients with homozygous CC, who had shorter latencies on unsuccessful Stop trials compared with healthy control participans of the same genotype (F_1,103_ = 3.703, *p* = 0.057). There was no evidence for post-error slowing, as reflected by RT following unsuccessful Stop trials, i.e. no significant main effects of genotype (F_2,110_ = 0.034, *p* = 0.967), diagnostic group (F_1,110_ = 0.001, *p* = 0.985), and no significant genotype-by-diagnosis interaction (F_2,110_ = 1.713, *p* = 0.185; Table 2). CUD patients showed a higher proportion of Go omissions compared with control participants (F_1,103_ = 8.53, *p* = 0.004), however, Go omissions were not significantly different between *rs36024* genotypes (F_2,110_ = 0.849, *p* = 0.431) and there was no genotype-by-diagnosis interaction (F_2,110_ = 1.734, *p* = 0.182, Table 2).

## Discussion

Our study aimed to elucidate putative genetic influences underlying addiction vulnerability. We used the stop-signal task to measure impaired self-control in CUD patients and found a significant interaction between the diagnosis of CUD and a genetic polymorphism that has been linked with addictive behavior. *rs36024* C-allele homozygotes in patients who were addicted to cocaine showed impaired response inhibition performance on the stop-signal task. This was reflected by prolonged SSRT and a lower stopping success rate compared with both C-allele homozygotes, who did not use cocaine, and their cocaine-addicted peers who were T-allele homozygotes. C-allele carriers (CT heterozygotes), who were addicted to cocaine, showed intermediate stopping performance. Our findings may thus present a genetic vulnerability marker which is not only important scientifically, but may in future also have clinical implications with respect to personalized addiction interventions.

### *rs36024* as a putative risk marker for cocaine addiction

The vulnerability to develop CUD is a complex non-Mendelian trait [5]. Genotype variation in over thirty genes has been associated with a diagnosis of CUD (Table S3). These polymorphisms appear to increase the risk of CUD either directly, or through interactions with environmental factors such as childhood adversity, as evidenced by genome-wide studies [47, 48]. While statistically powerful, the hypothesis-free nature of genome-wide searches means that the functional significance of the identified polymorphisms to addictive behavior remains unclear. This contrasts with candidate gene studies, which limit the analysis to gene products that are thought to modulate the brain reward circuitry and hypothesized to be disrupted in cocaine addiction. However, candidate gene analyses of stimulant drug addiction are generally challenging to replicate [49], although notable exceptions include single nucleotide polymorphisms in nicotinic acetylcholine receptor α_5_ subunit gene (*CHRNA5*) [50–54] and cannabinoid receptor gene (*CNR1*) [55, 56]. Relevant to the present study is the finding that the same risk allele in α_2A_-adrenergic receptor gene (*ADRA2A*) was implicated in impaired delay discounting of monetary rewards in cocaine users [57] and prolonged response inhibition in healthy volunteers on the stop-signal task [12]. This may suggest that genetic variation in the noradrenaline system in drug users and non- drug users could differentially modulate self-control abilities assessed by the stop-signal task - a candidate neurobehavioral endophenotype of stimulant drug addiction [58]. We decided to investigate the noradrenaline transporter *rs36024* single nucleotide polymorphism, which was highlighted in the largest study of response inhibition to date (Table S4 [15];) and we replicated these findings now in adult CUD patients.

### Noradrenergic modulation of self-control

Addiction has long been recognized as a disorder of impulse control [59–61], as reflected by maladaptive behaviors such as uncontrollable drug-seeking and taking. This loss of control over behavior is thought to arise from a failure of top-down inhibition of the subcortical nuclei by the prefrontal cortex [62, 63], which is itself under modulatory influence of noradrenergic projections from locus coeruleus [64]. Not only does increasing noradrenaline concentration modulate the activity of the fronto-striatal inhibitory network, it has also been shown to improve self-control in humans and animal models of impulsivity on the stop-signal task [18, 65–67] and a related test of motor impulsivity, the five-choice task [68–70]. Of particular relevance to addiction is the observation that stimulant drugs modulate the firing patterns of noradrenergic neurons [71]. Pharmacological enhancement of noradrenaline transmission is thus likely to alleviate impaired inhibition in CUD patients [72, 73]. Consistent with this idea, increasing extracellular noradrenaline concentrations and directly activating post-synaptic noradrenergic receptors improve stop-signal performance in CUD patients [26, 74] and healthy volunteers [66]. Stopping ability is thought to be mediated in part by a circuitry including the inferior frontal gyrus. Its activation during the stop-signal task correlates with noradrenaline availability [66], while the reduced white matter integrity has been associated with prolonged stopping responses in CUD patients and their unaffected siblings [7]. CUD patients and their healthy siblings shared not only the variability in prefrontal white matter, but also similar self-control impairments [7]. It is therefore tempting to speculate whether the unaffected siblings of CUD patients, who are at risk for developing CUD should they decide to use cocaine, might benefit from increased noradrenaline availability. Possibly, *rs36024* C-allelic variant represents a genetic biomarker, which underpins the observed familial vulnerability for cocaine addiction by altering noradrenaline action on the prefrontal-subcortical inhibitory system.

### Potential scientific and clinical implications

The interaction between the *rs36024* C-allele and the CUD diagnosis points towards pharmacogenetic effects influencing the efficacy of drugs that target the noradrenaline transporter in the treatment of addiction. However, atomoxetine is a relatively selective noradrenaline transporter inhibitor, which was found neither to reduce cocaine use in CUD patients [75], nor to improve their self-control [19]. Evidently, more studies in this area are warranted to evaluate the role of *rs36024* in noradrenaline transporter function, and the behavioral effects of atomoxetine in a randomized, placebo-controlled trial in C-allele-carrying CUD patients. Such potential for personalized pharmacological interventions in the treatment of addiction has been previously suggested for naltrexone in patients with alcohol use disorder carrying a risk allele in the opioid receptor gene [76]. Finally, our observation that only C-allele homozygotes with CUD diagnosis showed prolonged stop-signal reaction time might explain why several studies using the stop-signal task could not find response-inhibition impairments in CUD patients [77–82].

### Strengths and weaknesses

Our study has several strengths, including a validated, widely-used behavioral paradigm and well-characterized participants in terms of personality and demographic variables. Performance data were analyzed in line with the latest recommendations outlined by the SSRT consensus protocol [40]. The *rs36024* polymorphism in *SLC6A2* gene was selected on the basis of prior work [15] and does not seem to overlap with other known variants in this gene in our sample as reflected by nil linkage disequilibrium scores (NIH SNP Function Prediction database, https://snpinfo.niehs.nih.gov). We, however, acknowledge that it would have been desirable to replicate our findings in an endophenotype study, which includes not only CUD patients but also their first-degree relatives. Finally, we note that our study sample comprised predominantly male CUD patients, which reflects the male predominance of cocaine users in the UK population [83]. Future studies are recommended to increase the recruitment of female CUD patients. Future work may also want to expand our work on the *rs36024* single nucleotide polymorphism in abstinent CUD patients, who have likewise been shown to have prolonged SSRT [23].

## Conclusion

In summary, we provide the first demonstration of an interaction in stop-signal performance between a genetic polymorphism and a clinical diagnosis of CUD. Importantly, this genotype-by-CUD diagnosis interaction was not explained by comorbid opiate or cannabis dependence. A parsimonious explanation of our results favors the *rs36024* polymorphism acting as a genetic vulnerability marker, which may facilitate the transition from first cocaine use to addiction. Future pharmacological and neuroimaging studies in recreational cocaine users, CUD patients and their unaffected first-degree relatives would be needed to test this hypothesis.

## Supplementary information

Supplemental Material
