# Supplementary material for: Genotype-by-diagnosis interaction influences self-control in human cocaine addiction
Source: Transl Psychiatry. 2023 Feb 11;13:51. doi: 10.1038/s41398-023-02347-z (PMC9922269; doi:10.1038/s41398-023-02347-z)
Supplement: Supplementary file 1 — Supplemental Material [file 41398_2023_2347_MOESM1_ESM.pdf]

Supplemental Material

Genotype-by-diagnosis interaction influences self-control in human cocaine addiction

MM Graczyk, BJ Sahakian, TW Robbins, KD Ersche✉

✉ke220@cam.ac.uk

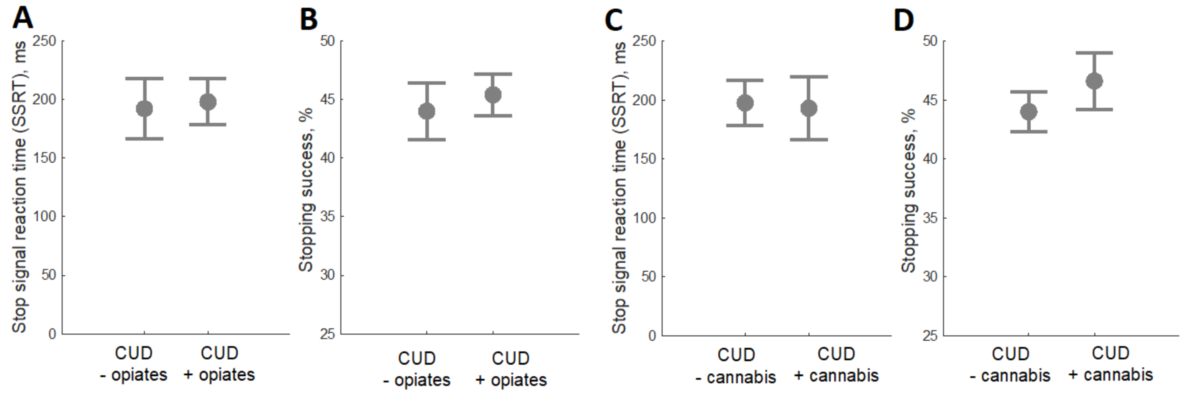

Figure S1: Stop-signal task performance of CUD patients with and without co-morbid opiate use disorder (**A** and **B**) as well as cannabis use disorder (**C** and **D**). Performance in these subgroups did not differ with respect to SSRT and the percentage of successful stops. [Error bars denote ±2 standard errors of the mean].

Table S1: Subgroup comparison of CUD patients with and without co-morbid opiate use disorder

	CUD - Opiates		CUD + Opiates		t	df	p
	Mean	Std.	Mean	Std.			
Sample size (n)	23	--	40	--	--	--	--
Age (years)	39.7	8.6	38.2	8.5	0.672	61	0.504
Genotype (CC, CT, TT)	8,8,7	--	17,14,9	--	0.581*	2	0.748
Verbal intelligence (NART score)	103.6	9.0	101.1	8.2	1.11	58	0.135
Impulsivity (BIS-11 total score)	75.2	9.8	75.5	9.7	-0.108	61	0.914
Childhood abuse (CTQ abuse score)	30.1	15.5	23.8	10.8	1.67	46	0.102
Duration of cocaine use (years)	15.5	8.7	16.0	5.6	-0.304	61	0.762

*chi-square statistic

Table S2: Subgroup comparison of CUD patients with and without co-morbid cannabis use disorder

	CUD - THC		CUD + THC		t	df	p
	Mean	Std.	Mean	Std.			
Sample size (n)	42	--	21	--	--	--	--
Age (years)	39.0	8.2	38.3	9.4	0.326	61	0.745
Genotype (CC, CT, TT)	17,17,8	--	8,5,8	--	3.13*	2	0.209
Verbal intelligence (NART score)	102.6	8.5	100.9	8.6	0.703	58	0.485
Impulsivity (BIS-11 total score)	75.0	10.6	76.1	7.5	-0.430	61	0.669
Childhood abuse (CTQ abuse score)	26.4	13.7	25.7	11.9	0.182	46	0.856
Duration of cocaine use (years)	15.3	6.4	16.9	7.6	-0.887	61	0.379

*chi-square statistic

Table S3. List of single nucleotide polymorphisms reported in genome-wide association and candidate gene studies to be associated with cocaine use.

SNP	Chr.	Gene	Association	Study	Publication
rs200085570	1	NA	CUD in EA	GWAS	[1]
rs2629540	10	FAM53B	CUD in AA and EA		
rs2456778	10	CDK1	CIP in AA		
rs150954431	12	NCOR2	CUD in EA		
rs61835088	1	FAM78B	CUD in AA and EA	GWAS	[2]
rs73404786	7	NA	CUD in EA		
rs149843442	1	LPHN2	Cocaine abuse in AA	GWAS	[3]
rs114492924	5	LINC01411	Cocaine abuse in AA		
rs6721393	2	LRP1B-KYNU	SUD in AA	GWAS	[4]
rs2394476	10	SLC25A16	SUD in AA		
rs1133503	6	MANEA	CIP in AA and EA	Low-density GWAS	[5]
rs1342872	1	NA	CUD in AA and EA		
rs1476880	4	NA			
rs1381355	4	MGC48628			
rs131020	22	NA			
rs597332	1	NTNG1	CUD in male French-Canadians	Candidate gene	[6]
rs503828					
rs678849	1	OPRD1	CUD in AA	Candidate gene	[7]
rs6736017	2	HTR2B	CUD in male French AC	Candidate gene	[8]
rs1978340	2	GAD1	White matter changes in CUD	Candidate gene	[9]
rs769390					
rs1803274	3	BChE	Crack cocaine use	Candidate gene	[10]
rs4263329	3	BChE	CUD in Brazilians	Candidate gene	[11]
rs6280	3	DRD3			
rs11503014	4	GABRA2	Childhood adversity and CUD risk	Candidate gene	[12]
rs6454674	6	CNR1	CUD in AA	Candidate gene	[13]
rs6454674			CUD in EA	Candidate gene	[14]
rs806368			CUD in AA and EA		
rs13005	6	HTR1B	CUD in EA and AA	Candidate gene	[15]
rs997917	8	OPRK1	CUD in AA	Candidate gene	[16]
rs10111937					
rs1342043	9	NCS1	CUD in male AA	Candidate gene	[17]
rs1020715	11	HTR3A	CUD in EA	Candidate gene	[18]
rs2229163	11	CHRM4	CUD in AA	Candidate gene	[19]
rs6561333	13	HTR2A	CUD in Europeans	Candidate gene	[20]
rs16969968	15	CHRNA5	Crack cocaine addiction	Candidate gene	[21]
rs588765	15				
rs16969968	15	CHRNA5	Crack cocaine addiction	Candidate gene	[22]
rs16969968	15	CHRNA5	CUD in AA	Candidate gene	[23]
rs684513	15				
rs16969968	15	CHRNA5	Personality and risk of CUD	Candidate gene	[24]
rs1437134	16	NFAT5	CUD in Spanish	Candidate gene	[25]
rs18321	17	NSF	CUD in Spanish	Candidate gene	[26]
rs1047383	20	PLCB1	CUD in Spanish	Candidate gene	[27]
rs910080	20	PDYN	CUD in AA and EA	Candidate gene	[28]

rs910079<br>rs2235749<br>rs737865<br>rs4680	22	COMT	CIP in AA<br>CIP in EA	Candidate gene	[29]

SNP: single nucleotide polymorphism; Chr.: chromosome; NA: not applicable; CUD: cocaine use disorder; CIP: cocaine-induced paranoia; SUD: stimulant use disorder; AA: African-American; EA: European-American; AC: African-Caribbean.

Table S4. List of single nucleotide polymorphisms reported in candidate gene studies to be associated with stop-signal task.

SNP	Chr.	Gene	Association	Study	Publication
rs2312955*<br>rs2229169*<br>rs4426564*<br>rs1168965*	2	ADRA2B	SST performance in Han Chinese	Candidate gene	[30]
rs2044081	4	GABRB1	Differential brain network activation in SST	Candidate gene	[31]
rs460000<br>rs37020	5	SLC6A3/ DAT1	SST performance in white Australians	Candidate gene	[32]
rs37020	5	SLC6A3/ DAT1	Differential brain network activation in SST	Candidate gene	[33]
rs1799971	6	OPRM1	Differential brain network activation in SST	Candidate gene	[34]
rs6296	6	HTR1B	Differential brain network activation in SST	Candidate gene	[35]
rs1800544	10	ADRA2A	SST performance in white Australians	Candidate gene	[36]
rs1800955	11	DRD4	SST performance in Spanish students	Candidate gene	[37]
rs4648317<br>rs12364283	11	DRD2	SST performance	Candidate gene	[38]
rs6277<br>rs6277	11	DRD2	SST performance in Dutch	Candidate gene	[39]<br>[40]
rs1800497	11	ANKK1	SST performance in male Spanish AUD	Candidate gene	[41]
rs1800497<br>rs6313	11<br>13	ANKK1<br>HTR2A	SST performance	Candidate gene	[42]
rs6313	13	HTR2A	SST performance in Polish AUD	Candidate gene	[43]
rs2242446<br>rs36024<br>rs187714<br>rs36023<br>rs16955591	16	SLC6A2	Right frontal network activation in SST	Candidate gene	[44]
rs1044396	20	CHRNA4	Differential brain network activation in SST	Candidate gene	[45]
rs4680	22	COMT	SST performance in Caucasians	Candidate gene	[46]
rs4680	22	COMT	Prefrontal network activation in males on SST	Candidate gene	[47]
rs4680	22	COMT	Differential brain network activation in SST	Candidate gene	[33]

SNP: single nucleotide polymorphism; Chr.: chromosome; SST: stop-signal task; AUD: alcohol use disorder.
*These SNPs were found to be associated as a haplotype

Supplemental References

1. Gelernter J, Sherva R, Koesterer R, Almasy L, Zhao H, Kranzler HR, et al. Genome-wide association study of cocaine dependence and related traits: FAM53B identified as a risk gene. Mol Psychiatry. 2014;19:717–723.
2. Sherva R, Zhu C, Wetherill L, Edenberg HJ, Johnson E, Degenhardt L, et al. Genome-wide association study of phenotypes measuring progression from first cocaine or opioid use to dependence reveals novel risk genes. Explor Med. 2021. 28 February 2021. https://doi.org/10.37349/emed.2021.00032.
3. Sun J, Kranzler HR, Gelernter J, Bi J. A genome-wide association study of cocaine use disorder accounting for phenotypic heterogeneity and gene–environment interaction. J Psychiatry Neurosci. 2020;45:34–44.
4. Cox J, Sherva R, Wetherill L, Foroud T, Edenberg HJ, Kranzler HR, et al. Genome-wide association study of stimulant dependence. Transl Psychiatry. 2021;11:363.
5. Yu Y, Kranzler HR, Panhuysen C, Weiss RD, Poling J, Farrer LA, et al. Substance dependence low-density whole genome association study in two distinct American populations. Hum Genet. 2008;123:495–506.
6. Kelaï S, Ramoz N, Moalic J-M, Noble F, Mechawar N, Imbeaud S, et al. Netrin G1: its downregulation in the nucleus accumbens of cocaine-conditioned mice and genetic association in human cocaine dependence: *NTNG1* and cocaine dependence. Addict Biol. 2018;23:448–460.
7. Crist RC, Ambrose-Lanci LM, Vaswani M, Clarke TK, Zeng A, Yuan C, et al. Case–control association analysis of polymorphisms in the delta-opioid receptor, OPRD1, with cocaine and opioid addicted populations. Drug Alcohol Depend. 2013;127:122–128.
8. Lacoste J, Lamy S, Ramoz N, Ballon N, Jehel L, Maroteaux L, et al. A positive association between a polymorphism in the *HTR2B* gene and cocaine-crack in a French Afro-Caribbean population. World J Biol Psychiatry. 2020;21:784–789.
9. Alballa T, Boone EL, Ma L, Snyder A, Moeller FG. Exploring the relationship between white matter integrity, cocaine use and GAD polymorphisms using Bayesian Model Averaging. PLOS ONE. 2021;16:e0254776.
10. Negrão AB, Pereira AC, Guindalini C, Santos HC, Messas GP, Laranjeira R, et al. Butyrylcholinesterase Genetic Variants: Association with Cocaine Dependence and Related Phenotypes. PLoS ONE. 2013;8:e80505.
11. Pego AMF, Leyton V, Miziara ID, Bortolin RH, Freitas RCC, Hirata M, et al. SNPs from BCHE and DRD3 genes associated to cocaine abuse amongst violent individuals from Sao Paulo, Brazil. Forensic Sci Int. 2020;317:110511.
12. Enoch M-A, Hodgkinson CA, Yuan Q, Shen P-H, Goldman D, Roy A. The Influence of GABRA2, Childhood Trauma, and Their Interaction on Alcohol, Heroin, and Cocaine Dependence. Biol Psychiatry. 2010;67:20–27.
13. Clarke T-K, Bloch PJ, Ambrose-Lanci LM, Ferraro TN, Berrettini WH, Kampman KM, et al. Further evidence for association of polymorphisms in the CNR1 gene with cocaine addiction: confirmation in an independent sample and meta-analysis: CNR1 and cocaine dependence. Addict Biol. 2013;18:702–708.
14. Zuo L, Kranzler HR, Luo X, Yang B, Weiss R, Brady K, et al. Interaction between Two Independent CNR1 Variants Increases Risk for Cocaine Dependence in European Americans: A Replication Study in Family-Based Sample and Population-Based Sample. Neuropsychopharmacology. 2009;34:1504–1513.
15. Cao J, LaRocque E, Li D. Associations of the 5-hydroxytryptamine (serotonin) Receptor 1B gene (HTR1B) with alcohol, cocaine, and heroin abuse. Am J Med Genet B Neuropsychiatr Genet. 2013;162:169–176.
16. Yuferov V, Butelman ER, Randesi M, Ott J, Kreek MJ. Analyses of polymorphisms of intron 2 of OPRK1 (kappa-opioid receptor gene) in association with opioid and cocaine dependence diagnoses in an African-American population. Neurosci Lett. 2022;768:136364.
17. Multani PK, Clarke T-K, Narasimhan S, Ambrose-Lanci L, Kampman KM, Pettinati HM, et al. Neuronal calcium sensor-1 and cocaine addiction: A genetic association study in African-Americans and European Americans. Neurosci Lett. 2012;531:46–51.
18. Yang J, Li MD. Association and interaction analyses of 5-HT3 receptor and serotonin transporter genes with alcohol, cocaine, and nicotine dependence using the SAGE data. Hum Genet. 2014;133:905–918.
19. Levran O, Randesi M, Peles E, Correa da Rosa J, Ott J, Rotrosen J, et al. African-specific variability in the acetylcholine muscarinic receptor M4: association with cocaine and heroin addiction. Pharmacogenomics. 2016;17:995–1003.
20. Fernàndez-Castillo N, Roncero C, Grau-Lopez L, Barral C, Prat G, Rodriguez-Cintas L, et al. Association study of 37 genes related to serotonin and dopamine neurotransmission and neurotrophic factors in cocaine dependence: Association of cocaine addiction and gene systems. Genes Brain Behav. 2013;12:39–46.
21. Aroche AP, Rovaris DL, Grevet EH, Stolf AR, Sanvicente-Vieira B, Kessler FHP, et al. Association of CHRNA5 Gene Variants with Crack Cocaine Addiction. NeuroMolecular Med. 2020;22:384–390.
22. Grucza RA, Wang JC, Stitzel JA, Hinrichs AL, Saccone SF, Saccone NL, et al. A Risk Allele for Nicotine Dependence in CHRNA5 Is a Protective Allele for Cocaine Dependence. Biol Psychiatry. 2008;64:922–929.

23. Sherva R, Kranzler HR, Yu Y, Logue MW, Poling J, Arias AJ, et al. Variation in Nicotinic Acetylcholine Receptor Genes is Associated with Multiple Substance Dependence Phenotypes. Neuropsychopharmacology. 2010;35:1921–1931.
24. Zayats T, Yang B-Z, Xie P, Poling J, Farrer LA, Gelernter J. A Complex Interplay between Personality Domains, Marital Status and a Variant in CHRNA5 on the Risks of Cocaine, Nicotine Dependences and Cocaine-Induced Paranoia. PLoS ONE. 2013;8:e49368.
25. Fernàndez-Castillo N, Cabana-Domínguez J, Soriano J, Sànchez-Mora C, Roncero C, Grau-López L, et al. Transcriptomic and genetic studies identify NFAT5 as a candidate gene for cocaine dependence. Transl Psychiatry. 2015;5:e667–e667.
26. Fernàndez-castillo N, Cormand B, Roncero C, Sánchez-Mora C, Grau-Lopez L, Gonzalvo B, et al. Candidate pathway association study in cocaine dependence: The control of neurotransmitter release. World J Biol Psychiatry. 2012;13:126–134.
27. Cabana-Domínguez J, Roncero C, Pineda-Cirera L, Palma-Álvarez RF, Ros-Cucurull E, Grau-López L, et al. Association of the PLCB1 gene with drug dependence. Sci Rep. 2017;7:10110.
28. Yuferov V, Ji F, Nielsen DA, Levran O, Ho A, Morgello S, et al. A Functional Haplotype Implicated in Vulnerability to Develop Cocaine Dependence is Associated with Reduced PDYN Expression in Human Brain. Neuropsychopharmacology. 2009;34:1185–1197.
29. Ittiwut R, Listman JB, Ittiwut C, Cubells JF, Weiss RD, Brady K, et al. Association between polymorphisms in catechol-O-methyltransferase (COMT) and cocaine-induced paranoia in European-American and African-American populations. Am J Med Genet B Neuropsychiatr Genet. 2011;156:651–660.
30. Lei X, Chen C, He Q, Moyzis R, Xue G, Chen C, et al. Haplotype Polymorphism in the Alpha-2B-Adrenergic Receptor Gene Influences Response Inhibition in a Large Chinese Sample. Neuropsychopharmacology. 2012;37:1115–1121.
31. Duka T, Nikolaou K, King SL, Banaschewski T, Bokde ALW, Büchel C, et al. GABRB1 Single Nucleotide Polymorphism Associated with Altered Brain Responses (but not Performance) during Measures of Impulsivity and Reward Sensitivity in Human Adolescents. Front Behav Neurosci. 2017;11.
32. Cummins TDR, Hawi Z, Hocking J, Strudwick M, Hester R, Garavan H, et al. Dopamine transporter genotype predicts behavioural and neural measures of response inhibition. Mol Psychiatry. 2012;17:1086–1092.
33. van Rooij D, Hoekstra PJ, Bralten J, Hakobjan M, Oosterlaan J, Franke B, et al. Influence of DAT1 and COMT variants on neural activation during response inhibition in adolescents with attention-deficit/hyperactivity disorder and healthy controls. Psychol Med. 2015;45:3159–3170.
34. Courtney KE, Ghahremani DG, Ray LA. Fronto-striatal functional connectivity during response inhibition in alcohol dependence: Inhibition and alcoholism. Addict Biol. 2013;18:593–604.
35. van Rooij D, Hartman CA, van Donkelaar MMJ, Bralten J, von Rhein D, Hakobjan M, et al. Variation in serotonin neurotransmission genes affects neural activation during response inhibition in adolescents and young adults with ADHD and healthy controls. World J Biol Psychiatry. 2015;16:625–634.
36. Cummins TDR, Jacoby O, Hawi Z, Nandam LS, Byrne MAV, Kim B-N, et al. Alpha-2A adrenergic receptor gene variants are associated with increased intra-individual variability in response time. Mol Psychiatry. 2014;19:1031–1036.
37. Kramer UM, Cunillera T, Camara E, Marco-Pallares J, Cucurell D, Nager W, et al. The Impact of Catechol-O-Methyltransferase and Dopamine D4 Receptor Genotypes on Neurophysiological Markers of Performance Monitoring. J Neurosci. 2007;27:14190–14198.
38. Hamidovic A, Dlugos A, Skol A, Palmer AA, de Wit H. Evaluation of genetic variability in the dopamine receptor D2 in relation to behavioral inhibition and impulsivity/sensation seeking: An exploratory study with d-amphetamine in healthy participants. Exp Clin Psychopharmacol. 2009;17:374–383.
39. Colzato LS, van den Wildenberg WPM, Van der Does AJW, Hommel B. Genetic markers of striatal dopamine predict individual differences in dysfunctional, but not functional impulsivity. Neuroscience. 2010;170:782–788.
40. Colzato LS, van den Wildenberg WPM, Hommel B. The genetic impact (C957T-DRD2) on inhibitory control is magnified by aging. Neuropsychologia. 2013;51:1377–1381.
41. Rodríguez-Jiménez R, Ávila C, Ponce G, Ibáñez MI, Rubio G, Jiménez-Arriero MA, et al. The Taq IA polymorphism linked to the DRD2 gene is related to lower attention and less inhibitory control in alcoholic patients. Eur Psychiatry. 2006;21:66–69.
42. Weafer J, Gray JC, Hernandez K, Palmer AA, MacKillop J, de Wit H. Hierarchical investigation of genetic influences on response inhibition in healthy young adults. Exp Clin Psychopharmacol. 2017;25:512–520.
43. Jakubczyk A, Wrzosek M, Łukaszkiewicz J, Sadowska-Mazuryk J, Matsumoto H, Śliwerska E, et al. The CC genotype in HTR2A T102C polymorphism is associated with behavioral impulsivity in alcohol-dependent patients. J Psychiatr Res. 2012;46:44–49.

44. Whelan R, Conrod PJ, Poline J-B, Lourdusamy A, Banaschewski T, Barker GJ, et al. Adolescent impulsivity phenotypes characterized by distinct brain networks. Nat Neurosci. 2012;15:920–925.
45. Sadaghiani S, Ng B, Altmann A, Poline J-B, Banaschewski T, Bokde ALW, et al. Overdominant Effect of a *CHRNA4* Polymorphism on Cingulo-Opercular Network Activity and Cognitive Control. J Neurosci. 2017;37:9657–9666.
46. Mione V, Canterini S, Brunamonti E, Pani P, Donno F, Fiorenza MT, et al. Both the COMT Val158Met single-nucleotide polymorphism and sex-dependent differences influence response inhibition. Front Behav Neurosci. 2015;9.
47. White TP, Loth E, Rubia K, Krabbendam L, Whelan R, Banaschewski T, et al. Sex Differences in COMT Polymorphism Effects on Prefrontal Inhibitory Control in Adolescence. Neuropsychopharmacology. 2014;39:2560–2569.
